# The impact of sewage effluents on water quality of Lake Hawassa, Ethiopia

**DOI:** 10.1186/s13065-023-00954-x

**Published:** 2023-04-26

**Authors:** Dessie Tibebe, Yemane Tesfaye, Yezbie Kassa

**Affiliations:** 1 Department of Chemistry, University of Gondar, P. Box 196, Gondar, Ethiopia; 2 Department of Biology, University of Gondar, P. Box 196, Gondar, Ethiopia

**Keywords:** Water quality, Physicochemical characteristics, HCA, PCA, LDA, Lake Hawassa

## Abstract

The physicochemical characteristics of water samples from Lake Hawassa was determined with the aim of pointing out possible impacts of industrial effluents, agricultural chemicals and domestic sewage on the water quality of the lake. For this, a total of 15 physicochemical parameters were measured in 72 water samples collected from four different locations on the lake that are adjacent to areas involved in various human activities including agriculture (Tikur Wuha), resort hotel (Haile Resort), public recreation (Gudumale) and referral hospital (Hitita). Samples were collected over a period of six months covering the dry and wet seasons in 2018/19. One-way analysis of variance revealed the presence of significant difference in the physicochemical quality of the lake’s water across the four study areas and the two seasons. Principal component analysis identified the most discriminating characteristics that differentiate the studied areas according to the nature and level of pollution status. Tikur Wuha area was found to be characterized by high levels of EC and TDS, the values of these parameters were about twice or more than that measured in the other areas. This was ascribed to contamination of the lake by runoff water from the surrounding farmlands. On the other hand, the water around the other three areas was characterized by high nitrate, sulfate and phosphate. Hierarchical cluster analysis classified the sampling areas in to two groups, where Tikur Wuha constituted one group and the other three locations the second group. Linear discriminant analysis provided 100% correct classification of the samples into the two cluster groups. The measured values of turbidity, fluoride and nitrate were found to be significantly higher than the standard limits set by national and international guidelines. These results show that the lake has been facing serious pollution problems from various anthropogenic activities.

## Introduction

A change in the water quality of a lake due to contamination negatively affects all levels of an ecosystem. It impacts the health of organisms at the lower level of the food chain as well as higher animals and humans. Deterioration of water quality can also lead to depletion of water resources and loss of aquatic biodiversity [1]. Adequate supply of potable water is necessary for the socio-economic development of a society. Fresh water resources all around the world are, however, under pressure from rapid industrialization, growing urbanization and increasing use of chemicals in agriculture [2]. The release of untreated and inadequately treated waste water onto water courses has both short and long term effect on the environment and human health. Fresh sources have been negatively impacted by waste water. Such impacts are dependent on the composition and concentration of the waste water contaminants as well as the volume and frequency of waste water effluents entering surface water sources [2].

The quality of water is highly important to understand the healthiness of a water body and its critical factor affecting human health and welfare [3, 4]. Water quality refers to the physical, chemical and biological characteristics of the water. Monitoring water quality is important in addressing water pollution problems through the formulation of suitable mitigation measures. Consequently, there has been an increasing demand for monitoring the quality of environmental waters by regular measurements of various quality parameters [5]. Among these, physicochemical characteristics of water are considered as important parameters that directly or indirectly affect water quality and consequently its sustainability [6–8]. Additionally, evaluation of water quality parameters against a standard guideline play important role in sustainable water resource management. Determining the quality of water is sensitive not just to the effects of an individual discharge, but to the combined effects of the whole range of different discharges into a water body. It enables an overall limit on levels of contaminants within a water body to be set according to the required uses of the water.

Lake Hawassa is one of the major rift valley lakes and fresh water resources in Ethiopia that is used for various purposes by semi-urban and urban dwellers. The lake plays important role in the livelihood of many people living in the region [9]. It is the source of commercial fishery. It is used as water supply for drinking and cultivation of vegetables by the surrounding communities. The lake also contributes to the economy of the country by being a tourist attraction point. Regardless of these, the lake has been subjected to pollutants from industrial effluents that are being released in to the city’s drainage system without proper treatment, which ultimately end up to the lake [10–13]. The lake has also been exposed to chemicals from neighboring agricultural activities [14–16]. Municipal waste water of Hawassa city has been and is being directly discharged into the lake. The indiscriminate disposal of waste water in to the lake can have an adverse impact on its sustainability and the associated ecosystem. This is mainly because untreated municipal waste water usually contains nutrients, such as nitrogen and phosphorus that stimulate the growth of aquatic plants to lead to eutrophication of the lake [17, 18]. The eutrophication process will alter basic characteristics of the lake such as depth, dissolved oxygen levels and water clarity [17]. Eutrophication may also create environmental conditions that favor the growth of toxin producing cyan bacteria, and exposure to such toxins is hazardous. Despite this, there is, generally, a lack of awareness among the local communities on the existing threat of permanent alteration of the lake influenced by increasing anthropogenic activities. Consequently, to minimize the effect of pollution through appropriate management strategy and legislation, determining the water quality of the lake is critical. This enhances evidence-based decision to ensure the sustainability of the lake and its ecosystem [17, 18, 24]. As a consequence, water pollution is currently a major environmental challenge at Lake Hawassa. Hence, multivariate statistical techniques have been widely adopted to analyze and evaluate surface and freshwater water quality, and are useful to verify temporal and spatial variations caused by natural and anthropogenic factors linked to seasonality [22–24]. Although the numerous management challenges, the multivariate techniques have a limited usage in the assessment of water quality in many lakes in developing countries including Lake Hawassa.

The aim of this study is to analyze the spatial and temporal difference of fifteen water quality parameters from the selected sampling sites of Lake Hawassa using multivariate analyses methods.

## Materials and methods

### Description of the study area

**Fig. 1:**
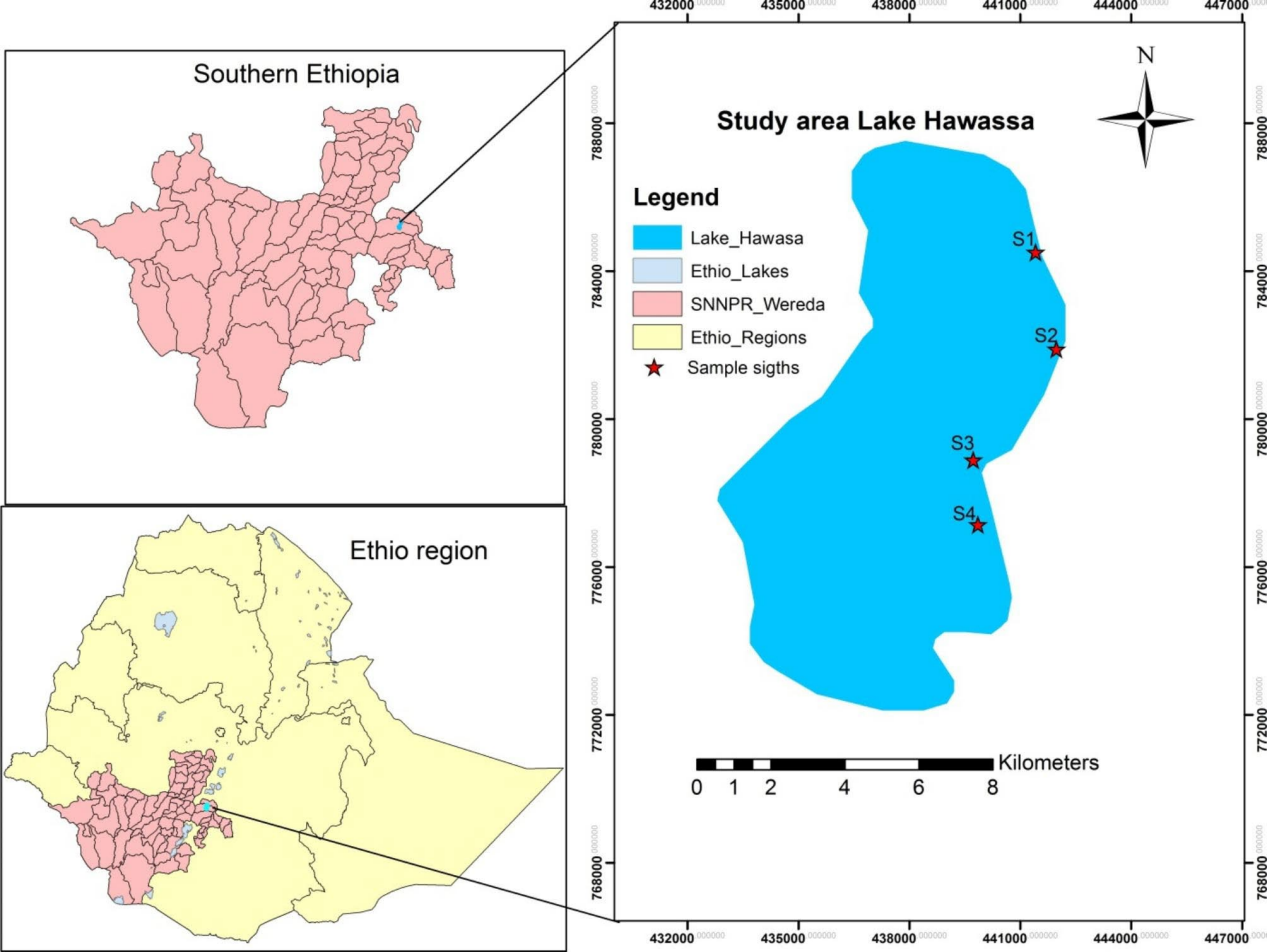
Map of study area. (Source: Wuletaw Mulualem)

Lake Hawassa is found adjacent to Hawassa city, Ethiopia (Fig. 1). The lake is located between 6°58’−7°14’N latitude and 35°22’−38°28’E longitude. The area receives a mean annual rainfall of 950 mm and air temperature of 19.8 °C. The catchment area of the lake is 1250 km^2^ located within a topographically closed basin that has no surface out flow. The lake receives surface inflow only from one perennial river, Tikur Wuha River.

### Sample collection

Water samples were collected from four different areas of the lake which are located adjacent to Tikur Wuha, Haile Resort, Gudumale and Hiteta localities of Hawassa city (Fig. 1). The sampling areas were selected based on the relative location (Table 1) and magnitude of human influence. The main anthropogenic activity around Tikur Wuha is agriculture, while Haile Resort is a popular resort in the city, Gudumale is a public recreational area and Hiteta hosts a major referral hospital in the city. Corresponding to each study area, one liter of water samples were collected from nine different sites. In order to assess seasonal variability, samples were collected on April, 10, 2019 in dry and July, 10, 2019. Accordingly, a total of 72 samples, with 36 samples from each season, were collected. Samples were collected at 50 cm depth from the surface of the lake and transferred to plastic bottles that were previously rinsed three times with the water to be sampled. Finally, the bottles were stored in insulated coolers containing ice and transported on the same day to the laboratory and kept in a refrigerator at 4 ^o^C until analysis.

**Table 1:** Geographical locations of the sampling areas on Lake Hawassa

Area	Latitude	Longitude
Tikur Wuha (S1)	038°28.867’	07°05.363’
Haile Resort (S2)	038°28.645’	07°04.776’
Gudumale (S3)	038°27.408’	07°02.487’
Hiteta (S4)	038°27.543’	07°01.544’

### Water sample analysis

The pH, EC, TDS, DO, turbidity and temperature were analyzed immediately in the field. For the analysis of chemical constituents, various standard methods used for the examination of surface waters were followed [19]. Samples were digested prior to the analysis. Briefly, a 20 mL mark was first made on a 100 mL beaker using a marker. A 100 mL of water was then poured in to the beaker and boiled until the solution reached up to the 20 mL mark. Finally 2 mL of nitric acid was added to get clear solution. All samples were analyzed in triplicates. Different instruments were employed in the analyses of the physicochemical characteristics of the water samples.

### Chemical analysis

The concentrations of all nutrients were determined for all samples following the standard procedures outlined in Palintest spectrophotometer 7100. Heavy metals (Cr, Cu, Fe) analyses were done by Atomic absorption spectrometry using acid digestion process.

### Multivariate statistical methods

#### Cluster analysis

CA classifies similar to the others in the cluster with respect to a predetermined selection criterion. Hierarchical agglomerative clustering is the most common approach, which provides intuitive similarity relationships between any one sample and the entire data set and is typically illustrated by a dendrogram. The dendrogram provides a visual summary of the clustering processes, presenting a picture of the groups and their proximity with a dramatic reduction in dimensionality of the original [24].

### Principal component analysis (PCA)

This technique was applied to recapitulate the statistical correlation among water quality parameters. The water quality parameter was consistent before PCA the analysis was performed in order to minimize the effect of different variables and their respective units of measurements [24].

### Data analysis

Data was analyzed using the statistical software package SPSS 20 (IBM Corp, USA). One-way analysis of variance (ANOVA) was used to compare the mean values of observations based on sampling areas and seasons. Differences in mean values were considered significant when p < 0.05. Multivariate statistical techniques of principal component analysis (PCA), hierarchical cluster analysis (HCA) and linear discriminate analysis (LDA) were also applied for data analysis.

## Results and discussion

### Spatial variation of water quality

All of the fifteen physicochemical parameters were measured in all of the water samples collected from the four areas studied (Table 2). Copper and sulfate were not detected in the dry season samples from Haile Resort and Tikur Wuha areas, respectively. One-way ANOVA revealed the presence of significant difference in the physicochemical quality of the lake’s water across the four study areas. In the dry season, the lake’s water adjacent to Tikur Wuha area was found to be significantly higher in EC (average 308 µS/cm) and TDS (average 154.7 mg/L) than the other areas, with average of 137.0−137.5 µS/cm and 87.9−98.3 mg/L, respectively. The measured values of EC and TDS in Tikur Wuha area were almost twice higher than that measured in the other areas. Tikur Wuha is located in the outskirts of Hawassa city where agriculture is predominant. Hence, the high concentration of dissolved substances, as reflected by the high EC and TDS values, may be ascribed to pollution of the lake by agricultural chemicals from the surrounding areas.

**Table 2:** The mean, maximum (Max), minimum (Min) and the associated standard deviation (SD) values corresponding to the various physicochemical parameters measured in water samples from four different areas of Lake Hawassa in dry seasons

Parameter		Sample Site				Parameter		Sample Site			
		Tikur Wuha	Haile Resort	Gudumale	Hiteta			Tikur Wuha	Haile Resort	Gudumale	Hiteta
EC	Mean	308.0	137	137.5	137.3	Cu	Mean	0.01	ND	0.32	0.034
	SD	0.9	0.9	0.4	0.1		SD	0.001	ND	0.02	0.001
	Min	307	136	137	137.2		Min	0.010	ND	0.31	0.031
	Max	309	138	138	137.4		Max	0.012	ND	0.34	0.035
TRB	Mean	4.3	21.5	10.8	18.2	K	Mean	27.7	26.0	9.2	26
	SD	0.01	0.43	0.1	0.1		SD	0.5	0.9	0.2	0.9
	Min	4.25	21	10.7	18.1		Min	27.0	25.0	9.0	25.0
	Max	4.28	22	10.9	18.3		Max	28.0	27.0	9.5	27.0
T	Mean	21.4	25.3	24.3	22.77	Fluoride	Mean	4.02	5.93	4.18	7.40
	SD	0.53	0.08	0.08	0.09		SD	0.005	0.1	0.013	0.087
	Min	21.0	25.2	24.2	22.7		Min	4.01	5.8	4.17	7.30
	Max	22.0	25.4	24.4	22.9		Max	4.02	6.00	4.20	7.50
TDS	Mean	154.7	87.9	88.3	98.3	Nitrite	Mean	0.007	0.006	0.015	0.005
	SD	1	0.1	0.09	1.3		SD	0.001	0.001	0.0001	0.0001
	Min	154	87.8	88.2	97.0		Min	0.006	0.005	0.014	0.005
	Max	156	88.0	88.4	100.0		Max	0.007	0.006	0.015	0.006
pH	Mean	7.1	9.2	9.2	9.1	Nitrate	Mean	1.63	2.17	80.3	39.3
	SD	0.08	0.1	0.07	0.07		SD	0.05	0.1	1.3	0.1
	Min	7.0	9.0	9.1	9.0		Min	1.6	2.1	79.0	39.2
	Max	7.2	9.3	9.3	9.2		Max	1.7	2.3	82.0	39.4
Fe	Mean	0.180	0.033	0.067	0.014	Sulfate	Mean	ND	1.46	17.3	7.33
	SD	0.009	0.002	0.005	0.001		SD	ND	0.05	0.5	0.5
	Min	0.17	0.031	0.06	0.014		Min	ND	1.4	17	7.0
	Max	0.19	0.035	0.07	0.015		Max	ND	1.5	18	8.0
Mn	Mean	0.20	0.40	0.67	0.67	Phosphate	Mean	0.12	0.19	0.24	0.28
	SD	0.006	0.01	0.05	0.05		SD	0.01	0.01	0.01	0.01
	Min	0.19	0.39	0.60	0.60		Min	0.11	0.18	0.23	0.28
	Max	0.21	0.41	0.70	0.70		Max	0.14	0.20	0.24	0.30
Cr	Mean	0.019	0.014	0.020	0.018						
	SD	0.001	0.001	0.001	0.001						
	Min	0.018	0.014	0.020	0.018						
	Max	0.020	0.015	0.021	0.019						

On the contrary, the water around Tikur Wuha was clearer during the dry season (average turbidity 4.3 NTU) than that of the other three sampling areas (average 10.8−21.5 NTU). The most turbid parts of the lake were found to be around Haile Resort (21.5 NTU) and Hitita (18.2 NTU). Furthermore, water samples from theses two areas contained significantly higher concentration of fluoride than that from the other areas. These may be due to waste from various human activities including recreational at Haile Resort, which also provides accommodation for people, and referral Hospital in case of Hiteta.

The other notable spatial variation was observed for nitrate and sulfate. The concentrations of nitrate and sulfate were high in water samples from Gudumale, 80.3 and 17.3 mg/L, respectively, and medium from Hiteta, 17.3 and 7.33 mg/L, respectively and insignificant from Tikur Wuha and Haile Resort areas. Furthermore, water samples from Gudumale and Hitita contained significantly higher phosphate than that from Tikur Wuha area. Besides the recreational activities that contaminate the lake with various wastes around Gudumale and discharge from the nearby hospital at Hitita, the high concentrations of nitrate, sulfate and phosphate may be from the untreated municipal wastes of Hawassa city, containing detergents and other chemicals, that enter in to the lake around these areas in which, the high concentrations of nitrate and phosphate may cause eutrophication of the lake ecosystem. On the other hand, Tikur Wuha is further from the main city and Haile Resort sampling area is adjacent to a privately owned resort hotel, hence the part of the lake around these two sampling areas is well protected from the city waste.

The concentrations of trace metals also varied significantly across the different studied parts of the lake. Particularly during the dry season, iron was significantly higher in Tikur Wuha than the other areas. The concentration of Fe found around Tikur Wuha was two to ten times higher than that found in water samples from the other areas. This may be due to leaching of iron rich soil in to the lake from the nearby farmlands at Tikur Wuha. On the other hand, the concentration of Cu was found to be significantly higher in the water samples from Gudumale, while Mn from Gudumale and Hiteta than from Tikur Wuha and Haile Resort areas. This is similar to the trend observed for nitrate, sulfate and phosphate, and hence it can be ascribed to contamination from untreated sewage effluents from the city and discharges from the nearby hospital.

In general, the lake’s water around Gudumale exhibited high concentrations of nitrate, sulfate, phosphate, Mn and Cu. This may be explained by, besides sewage effluents from the city, the excessive litter from the large number of people going for recreation to Gudumale, which is a popular public recreational area, where extensive food preparation and fishing activities take place.

#### Cluster analysis

Hierarchical cluster analysis (HCA) was applied to the analytical data set, corresponding to the dry season (Table 3), in order to explore the presence of identifiable groups of locations that have similar water quality status. The data set comprised 36 samples (9 sampling sites within each of the four areas) and 15 physicochemical properties measured (Table 4). Before HCA, the data was standardized using the z-scores method. The HCA was applied using the Euclidean distance as similarity measure with Ward’s method of linkage. The cluster analysis produced a two and three cluster solution that seems appropriate in the corresponding Dendrogram. However, examination of the plot of the distances against the number of clusters as well as the cluster centroids revealed that the sampling sites could be grouped into two significant clusters. Accordingly, three of the areas located around Haile Resort, Gudumale and Hiteta constitute one group, while Tikur Wuha the second.

**Table 3:** The physicochemical characteristics of water samples collected from four different areas (36 sites) of Lake Hawassa during the wet season (Temp. °C; pH, Turbidity, NTU; Electrical Conductivity, µS/cm; Total Dissolved Solids, mg/L; Nutrients (Sulfate, nitrate, nitrite, phosphate, fluoride), mg/L; Heavy metals (Fe, Mn, Cr, Cu, K), mg/L)

Area	EC	Turbidity	T	TDS	pH	Fe	Mn	Cr	Cu	K	Fluoride	Nitrite	Nitrate	Sulfate	Phosphate
Tikur Wuha	137.0	11.5	25.4	87.4	9.1	0.06	0.65	0.040	0.31	29.0	4.09	0.008	1.6	11.3	0.23
Tikur Wuha	137.0	11.9	25.3	87.5	9.2	0.08	0.65	0.020	0.34	29.5	4.09	0.007	1.7	11.2	0.24
Tikur Wuha	137.0	11.6	25.4	87.5	9.4	0.04	0.6	0.040	0.31	28.0	4.08	0.008	1.6	11.2	0.23
Tikur Wuha	138.0	11.5	25.4	87.9	9.3	0.07	0.4	0.020	0.33	29.0	4.08	0.006	1.7	11.3	0.24
Tikur Wuha	138.0	11.7	25.4	87.9	9.3	0.07	0.4	0.030	0.34	29.5	4.09	0.007	1.7	11.5	0.24
Tikur Wuha	136.0	11.8	25.3	88.5	9.3	0.08	0.7	0.030	0.33	29.5	4.09	0.008	1.7	11.5	0.23
Tikur Wuha	138.0	11.9	25.2	87.8	9.4	0.08	0.6	0.030	0.31	28.0	4.08	0.008	1.6	11.3	0.23
Tikur Wuha	136.5	11.5	25.2	87.6	9.1	0.05	0.6	0.035	0.32	28.6	4.07	0.006	1.5	11.1	0.21
Tikur Wuha	138.5	11.6	25.1	88.2	9.3	0.05	0.6	0.035	0.34	28.6	4.07	0.006	1.5	11.1	0.21
Haile Resort	137.9	23.2	24.2	88.2	9.3	0.07	0.5	0.021	0.31	9.0	5.18	0.020	78.0	17.3	0.25
Haile Resort	137.5	22.6	24.2	88.4	9.3	0.06	0.6	0.040	0.34	9.5	5.12	0.016	79.0	17.3	0.24
Haile Resort	137.6	23.0	24.4	88.3	9.2	0.07	0.6	0.040	0.31	9.3	5.12	0.020	80.0	18.0	0.24
Haile Resort	137.8	23.0	24.3	88.4	9.3	0.07	0.6	0.030	0.33	9.2	5.18	0.018	79.0	18.0	0.24
Haile Resort	137.8	23.0	24.3	88.4	9.3	0.07	0.7	0.030	0.33	9.3	5.18	0.015	79.0	18.0	0.24
Haile Resort	137.7	23.2	24.3	88.4	9.3	0.06	0.7	0.040	0.34	9.5	5.18	0.015	79.0	17.3	0.24
Haile Resort	137.5	21.0	24.2	88.3	9.3	0.06	0.7	0.020	0.34	9.5	5.11	0.017	79.0	17.6	0.23
Haile Resort	137.7	23.0	24.2	88.3	9.3	0.06	0.5	0.030	0.33	9.5	5.32	0.019	80.0	17.6	0.23
Haile Resort	137.8	23.0	24.3	88.3	9.2	0.06	0.6	0.020	0.34	9.5	5.31	0.019	80.0	17.9	0.23
Gudumale	137.9	11.9	24.2	88.2	9.3	0.06	0.6	0.020	0.34	9.2	4.18	0.011	89.0	19.0	0.23
Gudumale	137.5	11.9	24.2	88.4	9.3	0.06	0.6	0.030	0.32	9.1	4.20	0.017	89.0	17.0	0.24
Gudumale	137.7	12.3	24.4	88.3	9.1	0.05	0.6	0.020	0.31	9.1	4.17	0.012	90.0	17.0	0.24
Gudumale	137.7	12.2	24.3	88.3	9.1	0.05	0.7	0.020	0.32	9.1	4.19	0.016	89.0	20.0	0.24
Gudumale	137.7	12.2	24.3	88.3	9.1	0.07	0.7	0.020	0.32	9.2	4.19	0.019	90.0	20.0	0.24
Gudumale	137.8	11.9	24.3	88.3	9.1	0.08	0.7	0.030	0.32	9.2	4.20	0.018	90.0	18.0	0.24
Gudumale	137.9	11.9	24.4	88.4	9.4	0.06	0.6	0.030	0.32	9.1	4.19	0.012	90.0	18.0	0.23
Gudumale	137.8	12.2	24.4	88.4	9.2	0.06	0.5	0.030	0.32	9.3	4.20	0.027	89.0	17.0	0.23
Gudumale	137.8	12.2	23.4	88.3	9.1	0.07	0.5	0.030	0.31	9.3	4.20	0.016	87.0	17.0	0.24
Hiteta	137.2	19.1	22.7	98	9	0.013	0.8	0.021	0.03	26.0	7.40	0.005	39.2	5.0	0.27
Hiteta	137.2	19.3	22.9	100	9.2	0.016	0.6	0.017	0.04	25.0	7.50	0.004	39.4	7.0	0.38
Hiteta	137.2	19.1	22.7	99	9.1	0.015	0.6	0.017	0.03	25.0	7.30	0.006	39.2	8.0	0.28
Hiteta	137.1	19.1	22.7	99	9.1	0.014	0.7	0.018	0.03	26.0	7.50	0.005	39.4	6.0	0.26
Hiteta	137.1	19.3	22.8	98	9.1	0.016	0.8	0.022	0.03	23.0	7.50	0.005	39.4	8.0	0.26
Hiteta	137.2	19.3	22.8	98	9.1	0.014	0.5	0.029	0.03	23.0	7.50	0.004	39.3	5.0	0.26
Hiteta	137.2	19.1	22.8	98	9.1	0.017	0.6	0.015	0.03	23.0	7.40	0.004	39.3	5.0	0.24
Hiteta	137.2	19.1	23.2	99	9.1	0.015	0.6	0.018	0.04	27.0	7.40	0.006	39.2	6.0	0.26
Hiteta	137.1	19.3	23.2	100	9.2	0.017	0.7	0.019	0.04	27.0	7.30	0.006	39.3	6.0	0.28

**Table 4:** ANOVA Table obtained from the analysis of the variation of mean physicochemical characteristics between the two cluster groups. Difference is significant when *p* < 0.05

		Sum of Squares	df	Mean Square	F	p
EC	Between Groups	196812.85	1	196812.85	468383.37	0.00
	Within Groups	14.29	34	0.42		
Turbidity	Between Groups	1062.77	1	1062.77	66.32	0.00
	Within Groups	544.89	34	16.03		
T	Between Groups	48.27	1	48.27	51.50	0.00
	Within Groups	31.86	34	0.94		
TDS	Between Groups	26932.69	1	26932.69	1401.54	0.00
	Within Groups	653.36	34	19.22		
pH	Between Groups	29.66	1	29.66	3835.25	0.00
	Within Groups	0.26	34	0.01		
Fe	Between Groups	0.14	1	0.14	344.12	0.00
	Within Groups	0.01	34	0.00		
Mn	Between Groups	0.98	1	0.98	72.49	0.00
	Within Groups	0.46	34	0.01		
Cr	Between Groups	0.00	1	0.00	4.13	0.05
	Within Groups	0.00	34	0.00		
Cu	Between Groups	0.08	1	0.08	4.79	0.04
	Within Groups	0.56	34	0.02		
K	Between Groups	357.52	1	357.52	7.09	0.01
	Within Groups	1714.67	34	50.43		
Fluoride	Between Groups	22.41	1	22.41	16.28	0.00
	Within Groups	46.82	34	1.38		
Nitrite	Between Groups	0.00	1	0.00	1.54	0.22
	Within Groups	0.00	34	0.00		
Nitrate	Between Groups	10243.36	1	10243.36	12.65	0.00
	Within Groups	27532.91	34	809.79		
Sulfate	Between Groups	511.78	1	511.78	14.95	0.00
	Within Groups	1163.98	34	34.24		
Phosphate	Between Groups	0.09	1	0.09	70.11	0.00
	Within Groups	0.05	34	0.00		

In order to further validate the existence of cluster groups, one-way ANOVA was performed to test the presence of significant differences in the mean values of the physicochemical characteristics between the two groups (Table 4).

#### Principal component analysis

The application of principal component analysis (PCA) helps in the interpretation of complex data, examination of spatial patterns and identification of chemical species related to possible pollution sources that influence the lake’s water systems. For this, the data corresponding to the dry season was used. First, the suitability of the data set for the application of PCA was evaluated by using the Kaiser-Meyer-Olkin and Bartlett tests for the presence of significant (p < 0.05) correlation among the measured physicochemical characteristics. The results indicated the presence of strong (r = 0.648 to 0.995) and positive correlations between: conductivity with TDS and iron; turbidity with temperature and fluoride; TDS with iron; nitrate with sulfate; nitrite with nitrate and sulfate; manganese with nitrate, sulfate and phosphate; copper with nitrite, nitrate and sulfate, fluoride with phosphate. significant negative correlations were observed between: conductivity with turbidity, temperature, manganese and phosphate; turbidity with TDS, iron and chromium; temperature with TDS and iron; TDS with manganese and phosphate; iron with manganese, fluoride and phosphate; copper with potassium; potassium with nitrite, nitrate and sulfate. These negative and positive correlation of the different physicochemical parameters were attributable to their interaction, nature, seasonal variation and environmental factors.

**Fig. 2:**
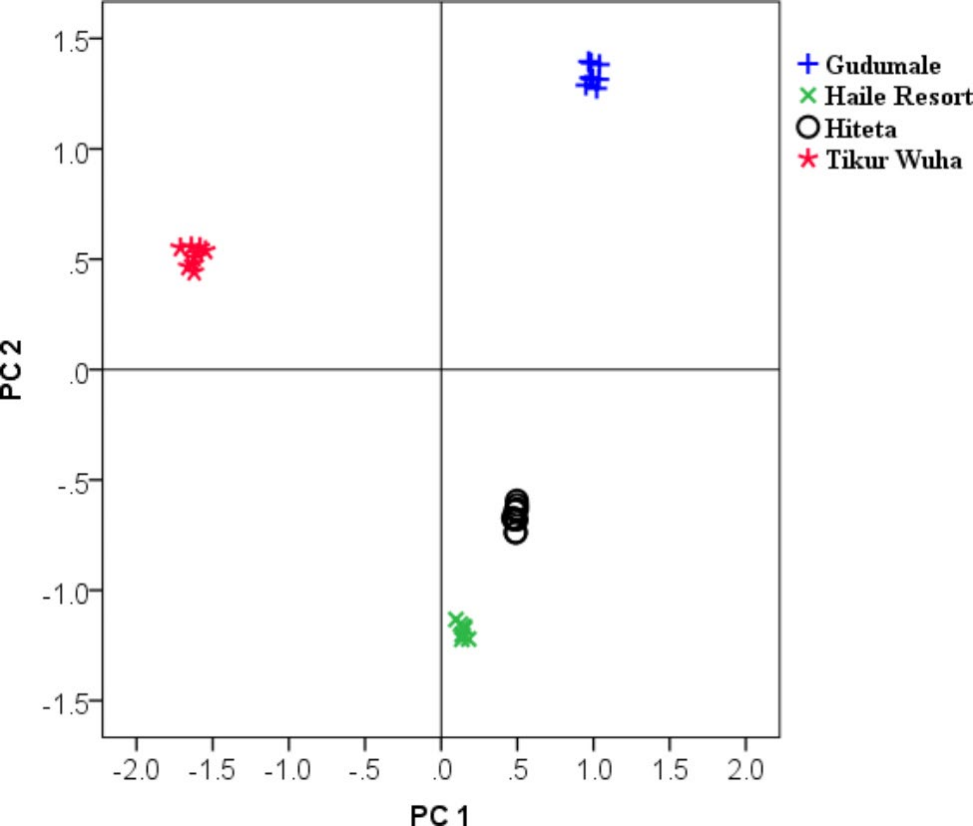
Scatter plot showing the distribution of samples on the space created by the scores of the first two principal components

In the PCA model, the first three principal components accounted for 99.0% of the total variance in the data set. The first component (PC1) explained 54.3% of the data variability, while the second (PC2) 34.6% and the third (PC3) 10.1%. Samples from the four areas of the lake show marked difference in their physicochemical qualities (Fig. 2). Similar to the previous observation with HCA, samples from Tikur Wuha area are clearly clustered in one group separated from samples of the other three areas by PC1, which explains the highest variation among samples.

**Fig. 3:**
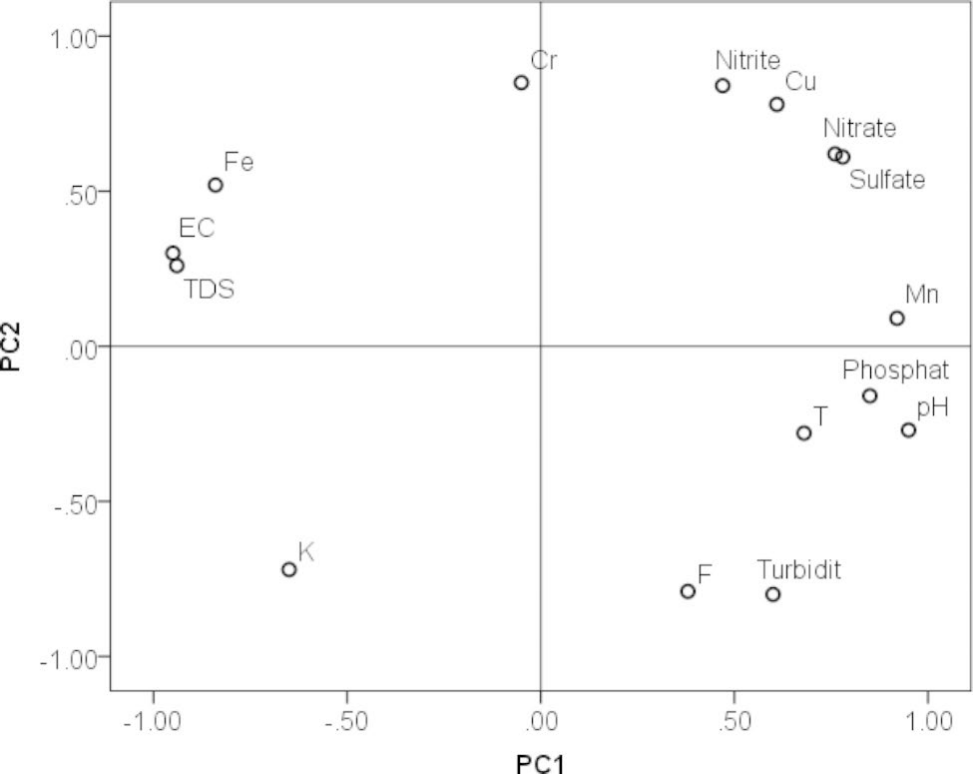
Scatter plot showing the correlation between the measured physicochemical parameters with the first two principal components

The loadings plot corresponding to the first two principal components is shown in Fig. 3. The first two PCs accounted for almost all of the information (90%) contained in the data. In order to simplify interpretation, only those parameters that are strongly correlated (|loading| > 0.75) with PC1 were considered. Accordingly, PC1 is strongly and negatively correlated with EC, TDS and Fe, while it is positively correlated with nitrate, sulfate, phosphate, Mn and pH. Examination of Figs. 2 and 3 reveals that the water in the part of the lake around Tikur Wuha area is best characterized by the high amounts of total dissolved materials, with high EC and TDS, and Fe. On the other hand, the water in the part of the lake around the other three areas, Gudumale, Haile Resort and Hitita, is high in nitrate, sulfate, phosphate, Mn and pH. Linear discriminant analysis based leave-one-out classification provided 100% correct classification of the samples into the two cluster groups. The first cluster constituted samples from Tikur Wuha area and the second from the other three areas.

On the other hand, with respect to PC2, the lake’s water around Gudumale is best characterized by higher levels of nitrate, sulfate and Cu, while around Haile Resort and Hitita by high levels of fluoride and turbidity. These observations are in agreement with the previous results obtained using ANOVA.

One-way ANOVA was also used to explore the presence of spatial variations in the mean values of the measured physicochemical parameters during the wet season (Table 5). Generally, the trend was found to be similar to that of the dry season. As was the case in the dry season, water samples from Tikur Wuha area, generally, contained lower levels of nitrate, phosphate, sulfate and manganese than the other areas. The most spatially varied characteristics of the water during the wet season were turbidity and TDS (Table 6). Application of HCA on data from the wet season classified the water samples in a slightly different way than the dry season. In the wet season samples tend to form three significant cluster groups, with samples from Tikur Wuha area constitute one cluster, Hiteta the second and Haile Resort and Gudumale together form the third cluster.

**Table 5:** Discriminant function coefficients

Parameter	Function
EC	−3.47
Turbidity	−1.03
T	−1.00
TDS	6.46
pH	−2.12
Fe	0.39
Mn	−0.36
Cr	0.80
Cu	9.57
K	3.47
Fluoride	−0.21
Nitrite	−0.10
Nitrate	−7.57
Sulfate	2.95
Phosphate	−0.19

**Table 6:** The mean, maximum (Max), minimum (Min) and the associated standard deviation (SD) values corresponding to the various physicochemical parameters measured in water samples from four different areas of Lake Hawassa in wet seasons

Parameter		Sample Site				Parameter		Sample Site			
		Tikur Wuha	Haile Resort	Gudumale	Hiteta			Tikur Wuha	Haile Resort	Gudumale	Hiteta
EC	Mean	137.3	137.7	137.8	137.2	Cu	Mean	0.326	0.330	0.320	0.033
	SD	0.8	0.1	0.1	0.1		SD	0.01	0.01	0.009	0.005
	Min	136.0	137.5	137.5	137.1		Min	0.31	0.31	0.31	0.03
	Max	138.5	137.9	137.9	137.2		Max	0.34	0.34	0.34	0.04
TRB	Mean	11.7	22.8	12.1	19.2	K	Mean	28.9	9.4	9.2	25.0
	SD	0.2	0.7	0.2	0.1		SD	0.6	0.2	0.08	1.7
	Min	11.5	21.0	11.9	19.1		Min	28.0	9.0	9.1	23.0
	Max	11.9	23.2	12.3	19.3		Max	29.5	9.5	9.3	27.0
T	Mean	25.3	24.3	24.2	22.9	Fluoride	Mean	4.08	5.19	4.19	7.42
	SD	0.1	0.1	0.3	0.2		SD	0.008	0.08	0.01	0.08
	Min	25.1	24.2	23.4	22.7		Min	4.07	5.11	4.17	7.30
	Max	25.4	24.4	24.4	23.2		Max	4.09	5.32	4.20	7.50
TDS	Mean	87.8	88.3	88.3	98.8	Nitrite	Mean	0.007	0.018	0.016	0.005
	SD	0.4	0.1	0.1	0.8		SD	0.001	0.002	0.005	0.001
	Min	87.4	88.2	88.2	98.0		Min	0.006	0.015	0.011	0.004
	Max	88.5	88.4	88.4	100		Max	0.008	0.020	0.027	0.006
pH	Mean	9.3	9.3	9.2	9.1	Nitrate	Mean	1.62	79.2	89.2	39.3
	SD	0.1	0.04	0.1	0.1		SD	0.08	0.7	1	0.09
	Min	9.1	9.2	9.1	9.0		Min	1.5	78	87	39.2
	Max	9.4	9.3	9.4	9.2		Max	1.7	80	90	39.4
Fe	Mean	0.064	0.064	0.062	0.015	Sulfate	Mean	11.3	17.7	18.1	6.2
	SD	0.01	0.005	0.01	0.001		SD	0.2	0.3	1.3	1.2
	Min	0.04	0.06	0.05	0.013		Min	11.1	17.3	17.0	5.0
	Max	0.08	0.07	0.08	0.017		Max	11.5	18.0	20.0	8.0
Mn	Mean	0.58	0.61	0.61	0.66	Phosphate	Mean	0.23	0.24	0.24	0.28
	SD	0.11	0.08	0.08	0.10		SD	0.01	0.01	0.01	0.04
	Min	0.4	0.5	0.5	0.5		Min	0.21	0.23	0.23	0.24
	Max	0.7	0.7	0.7	0.8		Max	0.24	0.25	0.24	0.38
Cr	Mean	0.031	0.030	0.026	0.020						
	SD	0.007	0.009	0.005	0.004						
	Min	0.02	0.02	0.02	0.015						
	Max	0.04	0.04	0.03	0.029						

Spatial variations in water quality between the two cluster groups, samples from Tikur Wuha as one cluster and samples from the other three locations as a second cluster, were further evaluated through linear discriminate analysis with the entire data set, comprising both dry and wet seasons. Discriminant analysis was used to find a linear combination of the observed data, called discriminant function that best separates the water samples in to the two clusters.

The relative contribution of each parameter to the discriminant function is given in Table 5. Parameters with the highest discriminating ability between the two clusters were copper, nitrate and TDS. Thus, these three parameters, also considering the previous results obtained with data from the dry season, can explain most of the spatial variations in the quality of water across the two seasons. The classification results showed that there are significant spatial differences between the two clusters with 100% of the samples correctly classified in to their respective clusters.

### Seasonal variations of water quality

One-way ANOVA was used to test the presence of significant differences in the mean values of the physicochemical qualities of the lake’s water between the dry and wet seasons. Accordingly, one significant observation was the decrease in EC and TDS and the increase in turbidity of the lake’s water around Tikur Wuha area during the wet season compared to the dry season. This is obvious as the decrease in EC and TDS can be explained from dilution of the lake from the higher run-off water during the rainy season that also caused more turbidity of the lake. Furthermore, the amounts of sulfate and phosphate increased dramatically at Tikur Wuha area during the wet season compared to the dry season. This might be due to the higher amount of flood water entering in to the lake during the rainy season that also brings with it the nutrients from farmlands surrounding Tikur Wuha area. On the other hand, the concentrations of nitrate and sulfate remain significantly higher in Gudumale than the other areas in both seasons.

#### Discriminant analysis

Seasonal variations in water quality were further evaluated through linear discriminant analysis (LDA). The analysis was used to find a linear combination of the observed data that best separate the water samples according to the quality parameters during the two seasons. The analysis was performed on the raw data after dividing the whole data set into the two seasonal groups. LDA was performed by excluding fluoride and Fe from the data set, as the concentration of these chemicals were significantly different between the two seasons as previously revealed by ANOVA. One discriminate function was calculated and Wilks’ Lambda test showed that the discriminant function is statistically significant (Table 7) and does better than chance at separating the seasons. Furthermore, 100% of the total variance between the seasons explained by the computed discriminant function.

**Table 7:** Wilks’ Lambda test of the discriminant function for seasonal variation of water quality

Wilks’ Lambda	Chi-square	df	Sig.
0.37	63.69	13	0.00

The relative contribution of each of the measured physicochemical water quality parameters to the discriminant function was assessed based on the absolute value of the discriminant function coefficients (Table 8). Parameters that showed the greatest discriminating ability between the two seasons were EC and TDS. The classification results showed the presence of significant differences between the two seasons and resulted in 89% correct classification of samples into their respective seasons.

**Table 8:** Standardized canonical discriminant function coefficients

Parameter	Function
EC	-6.29
Turbidity	1.21
TDS	4.85
Nitrate	3.56
Phosphate	-0.58
T	0.63
pH	-2.05
Mn	-0.53
Cr	0.38
Cu	3.59
K	2.79
Nitrite	-0.28
Sulfate	-2.18

### Comparison of the water quality of lake Hawassa with standards

The range of mean values of the physicochemical parameters measured in the water samples from Lake Hawassa across the different locations and seasons were compared with standard values used by some national and international guidelines (Table 9).

**Table 9:** Some guidelines showing standard limits for water samples

Parameters	[20] WHO (2004)^*a*^	[16] WHO (2005)^*b*^	[21] Ethiopia EPA (2009)^*a*^	[6] EPA (1994)^*c*^	This study
EC (µS/cm)	400		-	-	137–308
Turbidity (NTU)	5		5	-	4.3–22.8
T (^o^C)	25–30	-	-	-	21.4–25.3
TDS (mg/L)	500	80	1000	-	88–155
pH	6.5–8.5	6.0–9.0	6.5–8.5	-	7.1–9.3
Fe (mg/L)	0.3	-	0.3	-	0.015–0.180
Mn (mg/L)	0.4	-	0.5	-	0.20–0.67
Cr (mg/L)	0.01	-	0.05	-	0.014–0.031
Cu (mg/L)	1		2	-	0.01–0.33
K (mg/L)	20		1.5	-	9.2–28
Fluoride (mg/L)	1.5	-	1.5	-	4.0-7.4
Nitrite (mg/L)	3.0	-	3.0	-	0.007–0.018
Nitrate (mg/L)	50	50	50	13	1.6–89
Sulfate (mg/L)	250	-	250	-	1.4–18
Phosphate (mg/L)	0.02	< 1	-	0.01	0.12–0.28

Standard limit for drinking water; processed wastewater and domestic sewage discharges to surface water for general application; ^c^Lake water.

One significant deviation from the guideline values is the level of turbidity measured in the water samples. In all of the sampling areas, except in Tikur Wuha area during the dry season, turbidity of the lake’s water was significantly higher than the standard limits of WHO (2004) and Ethiopian Environmental Protection Agency (2009) for drinking water. Furthermore, turbidity increased during the wet season. The high extent of turbidity in the sampling areas indicates the extent of pollution of the lake by domestic sewage, as public waste is disposed in the city’s drainage system without proper management that ends up into the lake with flood water. Consequently, it can be concluded that the water is being polluted to the extent that it is unsafe for drinking purpose.

The second deviation from the guideline values, is the significantly higher concentration of fluoride found in the lake’s water from all the sampling areas and in both seasons. In addition to anthropogenic sources, this might be due to natural sources, as the lake is situated within the grate East African rift valley region, where high concentration of fluoride is common in water bodies in the region that is also a common cause of dental fluorosis among the communities.

The third deviation was the amount of nitrate found in most of the samples in levels of 3 to 6 times higher than that of the USEPA guideline for natural lake water. Furthermore, the amount of phosphate measured in samples from all the study areas was 10 times or more than the USEPA guideline for natural lake water. Both nitrate and phosphate tends to increase during the wet season, presumably due to runoff water during the rainy season that washed out fertilizers from neighboring farmlands and domestic waste containing detergents from city’s drainage system in to the lake. These results show that, beyond the immediate consequences of bad smell, the lake has been facing serious pollution problems that pose both environmental and health risks. This is because the continual release of untreated or inadequately treated sewage effluents, containing nutrients like phosphates and nitrates, in to the lake may lead to eutrophication. It will also creates environmental conditions that favor proliferation of water borne pathogens of toxin-producing cyan bacteria, and hence pose health risks to the large number of people and tourists going to the lake for recreation.

## Conclusion

Investigation of the impact of sewage effluents on the water quality of lake Hawassa obtained from the determination of the physicochemical parameters of the lake water quality, this study has revealed the following facts. One-way analysis of variance shown that presence of significant difference in the physicochemical quality of the lake’s water across the four study areas and the two seasons. Principal component analysis identified the most discriminating characteristics that differentiate the studied areas according to the nature and level of pollution status. Tikur Wuha area was found to be characterized by high levels of EC and TDS as compared to WHO, the values of these parameters were about twice or more than that measured in the other areas. On the other hand, the other three sampling sites were characterized by high nitrate, sulfate and phosphate. Hierarchical cluster analysis classified the sampling areas in to two groups, where Tikur Wuha constituted one group and the other three locations the second group. Linear discriminant analysis provided 100% correct classification of the samples into the two cluster groups. The measured values of turbidity, fluoride and nitrate were found to be significantly higher than the standard limits set by national and international guidelines. These results show that the lake has been facing serious pollution problems from various anthropogenic activities. From the result, most the analyzed water quality parameters in the surface water of the lake showed increased trend and these variables might be primarily due to different environmental factors associated with intensive anthropogenic activities in the lake catchment. The increasing trend in the studied water quality parameters may lead to long term ecological changes in the lake ecosystem unless possible measures should be taken.

## Data Availability

All data generated and analyzed are included within this research article.
